# Efficient Transient Transfection of Human Multiple Myeloma Cells by Electroporation – An Appraisal

**DOI:** 10.1371/journal.pone.0097443

**Published:** 2014-06-05

**Authors:** Torsten Steinbrunn, Manik Chatterjee, Ralf C. Bargou, Thorsten Stühmer

**Affiliations:** 1 Department of Internal Medicine II, Division of Hematology and Oncology, University Hospital of Würzburg, Würzburg, Germany; 2 Comprehensive Cancer Center Mainfranken, University Hospital of Würzburg, Würzburg, Germany; Meharry Medical College, United States of America

## Abstract

Cell lines represent the everyday workhorses for in vitro research on multiple myeloma (MM) and are regularly employed in all aspects of molecular and pharmacological investigations. Although loss-of-function studies using RNA interference in MM cell lines depend on successful knockdown, no well-established and widely applied protocol for efficient transient transfection has so far emerged. Here, we provide an appraisal of electroporation as a means to introduce either short-hairpin RNA expression vectors or synthesised siRNAs into MM cells. We found that electroporation using siRNAs was much more efficient than previously anticipated on the basis of transfection efficiencies deduced from EGFP-expression off protein expression vectors. Such knowledge can even confidently be exploited in “hard-to-transfect” MM cell lines to generate large numbers of transient knockdown phenotype MM cells. In addition, special attention was given to developing a protocol that provides easy implementation, good reproducibility and manageable experimental costs.

## Introduction

Multiple myeloma (MM) is a cancer affecting terminally differentiated plasma B cells [1]. MM accounts for about 15% of newly diagnosed hematologic cancers [2], [3] and the recent development of novel treatment options has led to considerably longer median survival [4]. While prolonged patient survival is being reported after the application of novel therapy regimens [5], [6], MM is generally still considered incurable with particularly unfavourable prognoses for certain genetically-defined patient subgroups [7], [8].

The profound advances in sequencing technologies now permit the use of primary MM cells to characterise an ever larger range of genetic traits throughout the course of a patient’s disease [9], [10], [11]. Nevertheless, human MM cell lines (HMCLs) are and will remain indispensable as tools for functional in vitro analyses and preclinical development of novel treatment approaches. Growing in suspension and/or semi-adherently, HMCLs do not count as particularly amenable to transient transfection with nucleic acids. Few publications have specifically addressed this topic [12], [13] and although a roster of anecdotal evidence implies various transient transfection methodologies for use with (specific) HMCLs [14], [15], [16], [17], [18], [19], [20], no broadly-used method of choice has so far emerged – not least, because transfection efficiency is usually either perceived as low or not easily determined in the first place. RNAi knockdown experiments in HMCLs can usefully complement pharmacologic inhibition studies and also offer a chance to target undruggable proteins. We have over the past ten years successfully used transient transfection of HMCLs with pSUPER short hairpin RNA expression vectors via electroporation [21], [22], [23], [24], [25]. To overcome the disadvantage of low transfection efficiencies we have applied a specific purification step, which leads to very pure fractions of strongly transfected cells [21], [23]. However, the necessity for purification adds to the amount of work-time needed, potentially increases the stressfulness of the whole methodology and also increases the overall cost of the procedure. Although this method can in principle be scaled up at will, it is in practice rather cumbersome to isolate high numbers (i.e. “millions”) of strongly transfected MM cells. We therefore tested the efficiency of knockdown approaches using the same electroporation conditions but employing siRNA or stealth siRNA oligonucleotides instead of short-hairpin expression vectors.

This manuscript describes in detail the procedures for plasmid versus oligonucleotide electroporation into HMCLs, compares the respective transfection and knockdown efficiencies and discusses the advantages and disadvantages of both experimental settings. Our aim is to summarise our experience with electroporation of MM cell lines that work well in our hands and to provide efficient models for functional analyses. We therefore explicitly intend to convey our personal take on all practical aspects connected to these tasks in order to provide solid guidance on how to plan, perform and interpret such experiments. Other points considered are the potential for easy application of these protocols in other laboratories, good feasibility of the procedures in the hands of researchers and technicians, and strict cost effectivity in order to serve as a workable standard procedure.

## Materials and Methods

### Human Multiple Myeloma Cell Lines (HMCLs)

HMCLs (AMO-1, JJN-3, L-363, OPM-2, RPMI-8228) were bought at the German Collection of Microorganisms and Cell Cultures (DSMZ; Braunschweig, Germany). INA-6 cells were a gift from Martin Gramatzki (University Medical Center Schleswig-Holstein, Kiel, Germany) [26]. After acquisition the cells were immediately expanded to create a stock bank of 50 vials stored in liquid nitrogen. One of these vials was then used to generate a working bank of between 30–50 vials. Every 3–4 months current cell cultures were retired and reinstated from the respective working banks (dead-end culture). Stock and working banks were confirmed to be negative for mycoplasma [27] at the time of their creation, and current cell cultures were also regularly tested. All cells were cultured at 5% CO_2_, 37°C, in RPMI-1640 medium supplemented with 10% FBS, 1 mM sodium pyruvate, 2 mM glutamine, and 100 U/ml penicillin +100 µg/ml streptomycin. INA-6 cells were supplied with 2 ng/ml recombinant human interleukin-6.

### Reagents

Annexin V was prepared according to the protocol detailed in [28], coupled to PromoFluor 647 using its commercially available N-hydroxysuccinimidyl ester (PromoCell, Heidelberg, Germany; PK-PF647-1), and the final concentration adjusted such that 1 µl produced a maximal signal shift in FACS measuremants of MM cells. Stealth siRNA against enhanced green fluorescent protein (cat. no. 12935-145) and custom-built stealth siRNA against human ERK2 (5′-GAGGAUUGAAGUAGAACAGGCUCUG-3′, equivalent to bases 900 to 924 of human *ERK2*) were obtained from Life Technologies (Darmstadt, Germany). 6-carboxyfluorescein (6-FAM)-labelled siRNA against ERK2 (5′-fluorescein-AAGAGGAUUGAAGUAGAACAG-dTdT-3′, a shorter version of the sequence mentioned above) was from Qiagen (Hilden, Germany). The pcDNA3.1-CD4Δ and the pSUPER-ERK2 vectors are described in [21].

### Electroporation of MM Cells

MM cells from routine cultures (cell densities 3×10^5^−7×10^5^/ml) were pelleted at 300×*g* and resuspended in fresh RPMI-1640 medium (i.e. freshly opened medium or medium stored at such conditions that preserve the pH of the unopened bottle [e.g. 15 ml screwcap tubes filled to the brim]) without additives. Cell densities in the final electroporation mix varied from between 2×10^7^/ml to 6×10^7^/ml, which for electroporations in 2 mm cuvettes (200 µl volume) represents a range from 0.4×10^7^−1.2×10^7^ cells per electroporation, and for 4 mm cuvettes (500 µl volume) translates to 1×10^7^−3×10^7^ cells per shot. Plasmid and/or siRNA solutions were dispensed into 1.5 ml Eppendorf tubes and mixed with the cell suspension by gentle pipetting. The complete range of electroporation mixes was prepared and electroporation carried out with a Gene Pulser (Bio-Rad Laboratories, München, Germany) at a capacity setting of 960 µF and with voltages ranging from 150 V-350 V. A single exponential decay pulse was applied and cell suspensions were immediately removed from the cuvette and pipetted into another tube containing 500 µl fresh medium without additives. Samples were left standing at room temperature until all electroporations were finished. Cells were then transferred to dishes with prewarmed full medium for further culture at standard conditions.

*Tip*: Re-use of electroporation cuvettes is permissible, at least when no use of electroporated cell material for PCR purposes is intended. We routinely use one cuvette for sequential performance of all electroporations within one experimental series, thoroughly rinsing it with PBS (squirt bottle) and quickly draining the cuvette (hitting it onto paper towels) in between electroporations. Afterwards the cuvette is cleaned with PBS and with EtOH and air-dried. This procedure can be repeated several times without significant loss of electroporation efficiency. Monitoring transfection efficiency by adding some pEGFP-N3 vector will help to establish a sensible routine.

Note: The use of really “fresh” medium in the cell suspension intended for electroporation was the most (in fact the only) critical factor for cell survival (at 280 V, a voltage that permitted the best transfection efficiency as measured by EGFP expression from pEGFP-N3) when we established this protocol for INA-6 cells (in contrast to such parameters as temperature before or after electroporation, recovery times, DNase-treatment after electroporation, cell density in culture or in the electroporation mix, presence of phenol red). This may reflect a particular sensitivity of INA-6 cells to the pH conditions of the solution after electroporation, and it need not necessarily be the same for other HMCLs. Because it is not much extra work we have simply extended this procedure to all HMCLs without further specific elaboration.

### Purification of Electroporated MM Cells

For simple removal of debris and dead cells the cell cultures at either day 1 or day 2 post-electroporation were pelleted and resuspended in a mixture of OptiPrep (a 60% solution of iodixanol; Progen Biotechnik, Heidelberg, Germany) with full medium (2.5 ml medium + 0.75 ml OptiPrep). The cell suspensions were overlayered with 200 µl PBS and centrifuged for 5 min at 4,000×*g*. Live cells, assembled at the interface between OptiPrep/medium and PBS, were once washed with full medium and taken into culture for further use in experiments.

For the column purification procedure using MACSelect 4 MicroBeads (Miltenyi Biotech, Bergisch-Gladbach, Germany) the cell cultures at either day 1 or day 2 post-electroporation were first washed with PBS and then with cold column buffer (PBS with 0.5% FBS and 2.5 mM EDTA). After resuspension in 340 µl cold column buffer 60 µl CD4 MicroBeads (Miltenyi Biotech, cat. no. 130-070-101) were added, and the mixture was incubated for 10–20 min at 4°C with occasional flicking. Samples were run over paramagnetic bead-filled columns (Large cell columns, Miltenyi Biotech, cat. no. 130-042-202), these were washed with cold column buffer, removed from the magnet and the retained cells flushed out with 2 ml full medium. Because the column run also retains significant numbers of dead cells it is necessary to subsequently subject the eluate to the above-described OptiPrep purification procedure.

Note: It is not useful to perform dead cell removal before the column run, because the column procedure will re-concentrate the small amount of debris that is still present and the end result is much dirtier.

*Tip*: The paramagnetic bead columns can be re-used. For regeneration we flush them 3–4 times with hot tap water, then once with distilled water (to remove salts) and finally with 96% ethanol (to remove most of the water). Columns are then quickly dried in an incubator at up to 65°C (reasonably quick drying is necessary because otherwise the columns turn rusty and should be discarded). To re-use these columns, 100 µl 80% ethanol is applied in order to ensure good wetting of the beads and complete air removal from the column before the new sample is loaded.

*Tip*: In order to restrict the flow rate through the Large cell columns the company provides flow resistors (i.e. 23 gauge hypodermic needles) with each box of columns. In our experience and for reasons entirely unclear the use of 23 gauge needles from Terumo (Neolus, NN-2332R) results in considerably more consistent sample flow and less occasions of complete flow blockage.

### Western Blotting and Antibodies

Frozen cell pellets were dissolved in Laemmlie-buffer (60 mM Tris-HCl, 10% glycerol, 2% SDS, 10% β-mercaptoethanol, 0.01% bromophenol blue; pH 6.8) (10 µl buffer per 100,000 cells) and subjected to sonication (3–5 s on ice with a UP50H sonicator equipped with an MS1 sonotrode) (Hielscher, Teltow, Germany). Samples were then heated to 89°C for 3 min, spun for 5 min at room temperature and the supernatants used for standard SDS-PAGE with 12% gels. Wet blotting was carried out in Mini Trans-Blot modules (Bio-Rad Laboratories) using nitrocellulose membranes and blotting buffer (20% v/v methanol, 25 mM Tris-HCl, 192 mM glycine, pH 8.6). The following antibodies were used for target detection: anti-ERK1/2 (Cell Signaling Technology, Frankfurt am Main, Germany; no. 9102), anti-ERK1/2 (Santa Cruz Biotechnology, Heidelberg, Germany; sc-94), anti-phospho-ERK1/2 (Cell Signaling Technology; no. 9101), anti-tubulin (Biozol, Eching, Germany; BZL03568). The secondary antibodies were from Jackson ImmunoResearch Laboratories (Newmarket, UK): anti-rat-HRP (112-036-062), anti-rabbit HRP (111-036-045). A freshly made solution of luminol (2.5 mM), p-coumaric acid (0.2 mM) and H_2_O_2_ (0.01%) in 100 mM Tris-HCl (pH 8.8) was used for chemiluminescent detection [29].

### Flow Cytometry

Cells were washed with PBS, pelleted and resuspended in 200 µl of cold annexin V binding buffer (10 mM HEPES/NaOH, 140 mM NaCl, 2.5 mM CaCl_2_; pH 7.4) containing 1 µl of annexin V-PromoFluor 647 solution (see Reagents) and 1 µg/ml propidium iodide. Flow cytometry was performed using a FACSCalibur (BD Biosciences, Heidelberg, Germany). Datafiles were analysed with FlowJo version 8.8.7 (Tree Star, Inc., Ashland, U.S.A.).

### Statistical Analysis

Calculation of the “range overlap” in FACS-based measurements for a given time-point: Measured events were gated for the live cell population according to the forward/sideward scatter pattern. The signal range containing the central 98% of events for the control sample measurement (i.e. all events between the first and the 99^th^ percentile) was then determined. Finally, it was calculated what percentage of the total events measured for the targeted sample fell into this range.

## Results and Discussion

### Electroporation of HMCLs with Expression Plasmids Followed by Cell Separation Yields Pure Fractions of Strongly Transfected Cells

**Figure 1:**
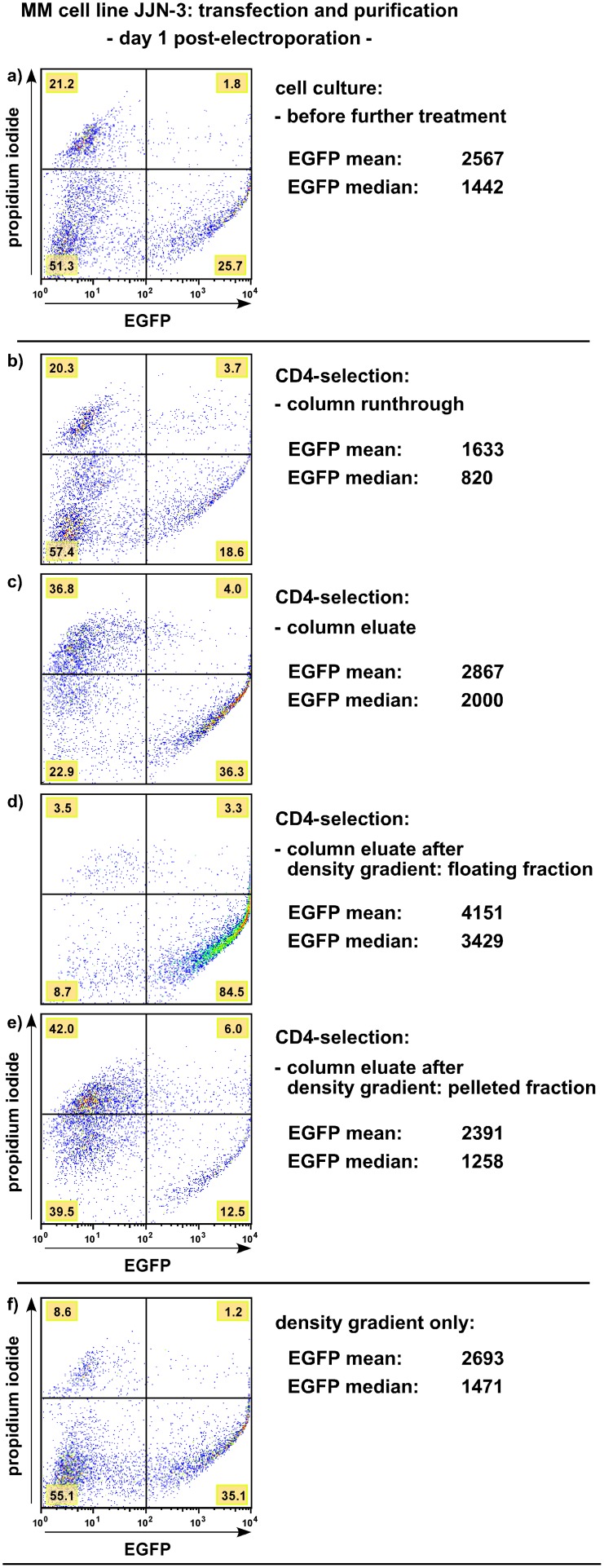
Electroporation of MM cell lines and subsequent purification of transfected cells. Shown is a representative example of the procedure using the well-transfectable MM cell line JJN-3. This standard column purification has now been performed hundreds of times in our laboratory and is also easily applicable for MM cell lines INA-6, KMS-11, L-363, MM.1S and U-266 (Table 1). a) Cell culture one day after electroporation with expression plasmids for enhanced green fluorescent protein (EGFP) and CD4Δ, showing about a quarter of cells strongly positive for EGFP. b)-e) Enrichment of strongly transfected cells by selection for CD4 surface expression (CD4 MicroBead column selection). b) Column runthrough of cell culture shown in a). Of note is the similar look with a), but with depletion of the strongest transfected cells in b). c) Column eluate of the cell culture shown in a). Untransfected cells (EGFP- and CD4Δ-negative) have effectively been removed, but the column procedure tends to retain significant amounts of dead cells (EGFP-negative, PI-positive). d) Floating fraction of the column eluate as shown in c) after “density gradient” (more properly: density step) treatment using OptiPrep, consisting mostly of viable and strongly transfected cells. e) Pelleted fraction of the column eluate as shown in c) after “density gradient” treatment using OptiPrep, consisting mostly of debris. f) Removal of debris by OptiPrep treatment from the cell culture as shown in a) without prior column separation, leaving two main fractions which are either EGFP-negative, or distinctly EGFP-positive. See Methods section for further details.

In order to obtain homogeneous fractions of transiently transfected MM cells we have established a protocol that combines electroporation with subsequent isolation of the most strongly affected cells (Fig. 1). This methodology allows very good knockdown efficiencies with pSUPER-based short-hairpin RNA expression vectors (applied, for example, in [21], [23], [24], [25]). Transfection efficiency is judged by introduction of an expression plasmid for EGFP (Fig. 1a), and – dependent on the HMCL – transfection efficiencies of up to 40% can be achieved with standard gear electroporations (Table 1). However, the large fraction of EGFP-negative cells represents an undesired background which we have sought to eliminate. Either cell sorting for EGFP-positive cells (not shown), or co-electroporation of an expression plasmid for CD4Δ [21] and subsequent microbead selection of CD4-positive cells (exemplarily shown in Fig. 1b–e) can be employed to obtain highly enriched fractions of strongly transfected cells (Fig. 1d, see Methods section for an exact description of the purification steps). These cells can then be used in experiments involving their actual payloads, for example co-transfected shRNA expression vectors [21], [23], [24], [25], and they are suitable for transient knockdown studies in applications such as apoptosis induction, drug testing, proliferation assessments or Western blotting. Furthermore, the transfection procedure itself is very cheap and permits easy scaling-up, because once effective shRNA-expression plasmids have been generated they can be produced at will and at very low cost. On the downside, FACS-based cell isolation necessitates access to such a service, potentially incurring fees and compelling researchers to abide by the operator’s schedule. The microbead purification approach has the advantage that it is fast and that all steps can easily be performed in the laboratory. However, this protocol relies on an expensive reagent (CD4 microbeads), and because the microbead columns also retain dead MM cells (Fig. 1c) a density gradient centrifugation step that removes this debris is required (Fig. 1d). Last, not every HMCL seems able to implement extracellular presentation of CD4Δ (most notably cell line AMO-1; TStü, personal observation), rendering such cell lines unsuitable for microbead purification protocols.

**Table 1:** Parameters for MM cell line electroporation.

HMCL	Optimal voltage rangewith 4 mm cuvette,max. 5×10^7^ cells/ml	Optimal voltage rangewith 2 mm cuvette,max. 2.5×10^7^ cells/ml	Range of transfected cells using pEGFP-N3
AMO-1	270–300 V	170–180 V	20–30%
INA-6	270–280 V	170–180 V	20–30%
JJN-3	270–300 V	170–180 V	20–30%
KMS-11	270–300 V	180–200 V	20–30%
KMS-12-BM	270–300 V	180–200 V	20–30%
L-363	270–300 V	170–180 V	20–30%
MM.1S	300–320 V	180–200 V	20–30%
MOLP-8	260–270 V	none	10–20%
NCI-H929	240–250 V	none	<10%
OPM-2	240–250 V	170–200 V	<10%
RPMI-8226	270–300 V	160–180 V	<10%
U-266	270–300 V	220–240 V	10–20%

Rule-of-thumb voltage settings for different HMCLs as deduced from electroporation with an expression plasmid for enhanced green fluorescent protein (pEGFP-N3). All transfections were carried out with a Gene Pulser (Bio-Rad) with a capacity setting of 960 µF. The whole unpurified cell culture was measured (FACS) at day one post-electroporation. The percentage ranges given are to be considered as guidelines for standard results based on between 10 (cell lines not regularly used in our experiments) and hundreds of electroporations (the regularly used “well-transfectable” MM lines). The best electroporations achieved have yielded up to 40% EGFP-positive cells, but suboptimal conditions - for example due to overly high cell densities in the preceding cell culture - may result in lower efficiencies than indicated.

### Electroporation of HMCLs with siRNA Oligonucleotides Results in Homogeneous Transfection of the Whole Viable Cell Population

Using commercial siRNA oligonucleotides instead of shRNA expression plasmids in electroporations is considerably more expensive. However, a much larger number of functional siRNA-oligonucleotide sequences have been published, and siRNA-oligonucleotide pools provide the fastest way to assess molecular and biological effects of target knockdown when only trial experiments or a limited set of electroporations are required. We therefore decided to rigorously test if our protocol could be adapted to perform (cost-) effective siRNA oligonucleotide-mediated knockdown studies in MM cells. The central question was whether the essentially EGFP-negative cell fraction (for example, the 55.1% of cells logged in the lower left quadrant in the JJN-3 electroporation shown in Fig. 1f) might nevertheless be accessible to the much smaller RNA oligonucleotides, and whether any such effects are strong enough to result in substantial and reliable target depletion.

**Figure 2:**
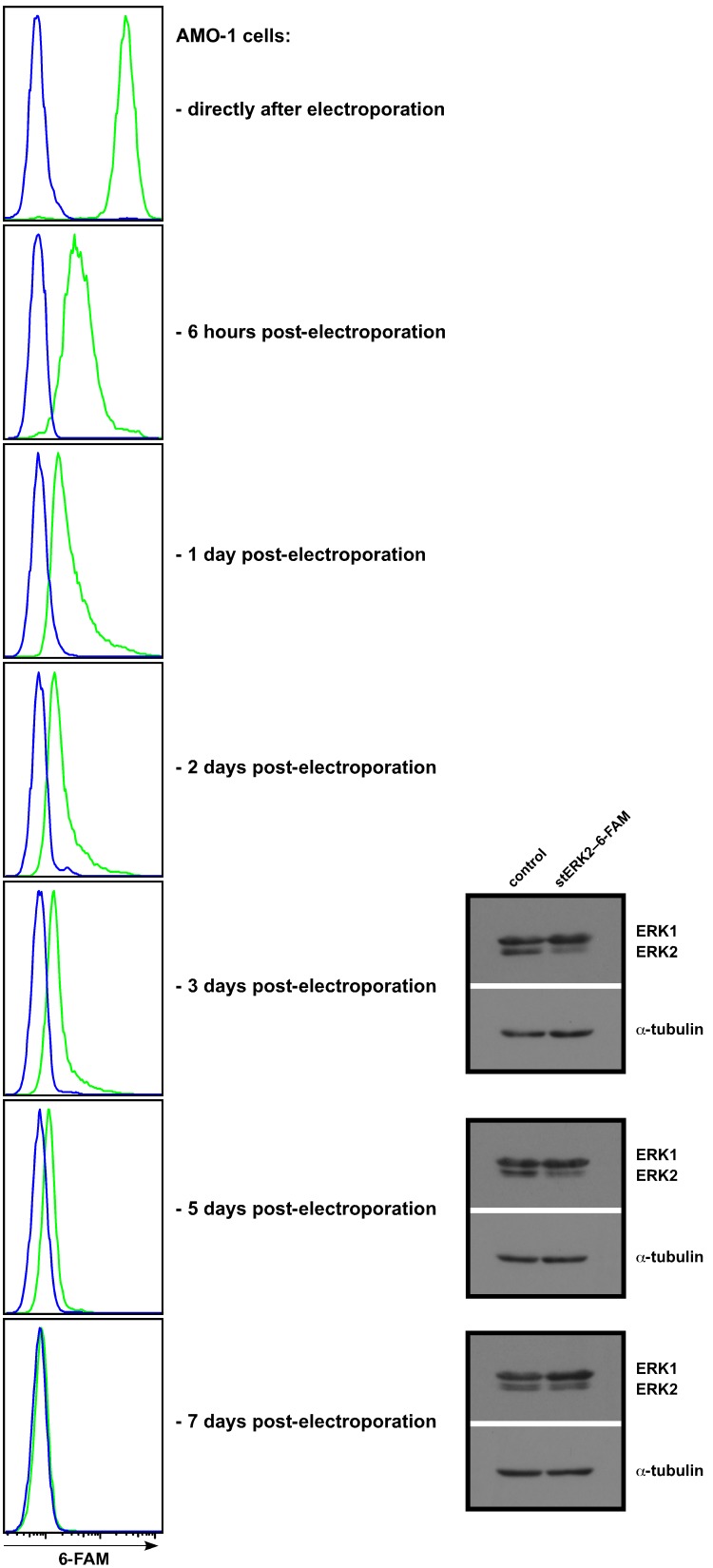
Electroporation of AMO-1 cells with a 6-FAM-labelled siRNA oligonucleotide. Left column: Fluorescence of AMO-1 cells electroporated with the siERK2-6-FAM oligonucleotide (green curve) in relation to mock transfected cells (blue curve) at different time points post-electroporation. Right: Western analysis for ERK2 knockdown at days 3, 5, 7 post-electroporation. One representative experiment from a total of three is shown. Anti-ERK1/2 antibody: CST.

We first employed an siRNA against human extracellular signal-regulated kinase 2 (ERK2) coupled to the fluorescent dye 6-carboxyfluorescein (6-FAM) to monitor transfection and knockdown efficiency in MM cell lines AMO-1 and RPMI-8226 (Fig. 2 and Fig. S1). ERK2 was chosen as a target because specific protein depletion in relation to its homolog, ERK1, is readily detectable by Western blotting with a single antibody, and because single ERK isoform knockdown has no adverse effect on the survival or proliferation of MM cells (TSts, personal observations). The siRNA sequence was based on the same sequence that had been found effective when used within an shRNA-expression vector [21]. Shortly after electroporation with siERK2-6-FAM the whole viable MM cell population showed a strong shift in fluorescence (for example: a 43-fold shift of the median in the exemplary experiment shown for AMO-1 cells in Fig. 2; a 25-fold shift of the median in the exemplary experiment shown for RPMI-8226 in Fig. S1), which within 24 h quickly declined to lower, although still clearly and universally detectable, levels. This was followed by a slower decline in fluorescence intensity, until at days 6–7 the signal was no longer detectable (Fig. 2). The range overlap (see Statistical analysis) for 6-FAM-labelled siRNA transfected cells with control siRNA transfected cells was just 1.3%±0.35% (n = 3) for AMO-1 cells at the 0 h timepoint, indicating that initially virtually the whole cell population had acquired the labelled siRNA. Although Western blotting of cells harvested at days 3, 5 and 7 post-electroporation confirmed reduced ERK2 levels at early timepoints in the siERK2-6-FAM treated fraction (Fig. 2; Fig. S1), significant quantities of the protein still remained. These experiments were therefore inconclusive: the universal shift in fluorescence and its subsequent fast decline could represent a genuine effect showing efficient introduction of the labelled siRNA into MM cells with subsequent fast processing in the RNA-induced silencing complex. However, the mediocre knockdown efficiency would then have to be explained by poor siRNA performance, possibly due to the presence of the fluorescent label. Alternatively, if the initial strong shift in fluorescence intensity was mainly artefactual, caused, for example, by labelled siRNA sticking to the cell surface after electroporation, then the moderate ERK2 knockdown might have been the result of inefficient or random introduction of the siRNA into the MM cell population.

**Figure 3:**
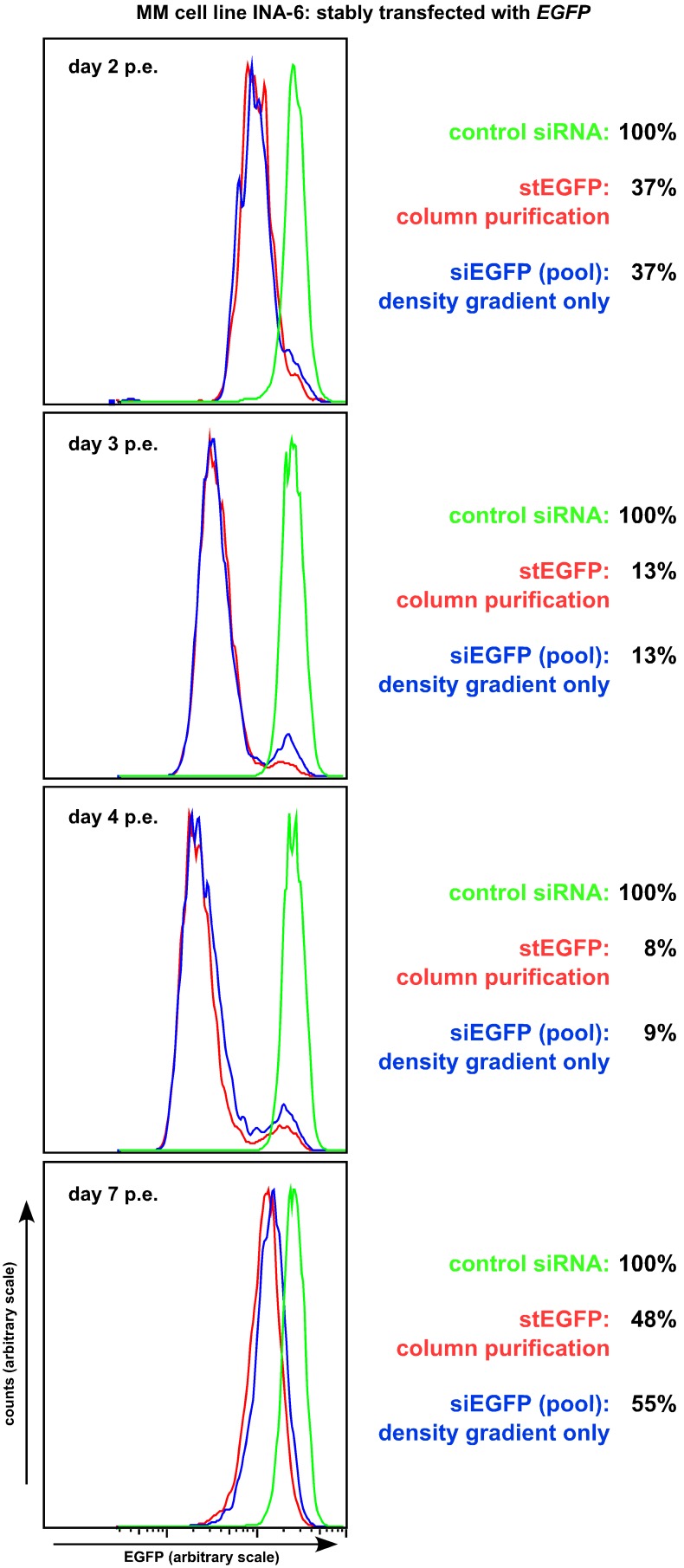
Electroporation of INA-6 cells stably expressing enhanced green fluorescent protein with an siRNA oligonucleotide against EGFP. INA-6-EGFP cells were electroporated with a solution containing a stealth siRNA targeting EGFP as well as an expression plasmid for CD4Δ. One day post-electroporation one half of the cell culture was purified according to the column procedure (red curves, also see Fig. 1b)–e)), whereas the other half only underwent debris removal with OptiPrep (blue curves, also see Fig. 1f)). Purified cells were further cultured and FACS-analysed for EGFP expression at the times indicated. Only the live cell fraction (as demarcated in the forward/sideward scatter) was analysed and plotted against similarly treated INA-6-EGFP cells (green curves) transfected with a non-EGFP targeting siRNA. Knockdown efficiency was essentially identical in strength and over time between both purification approaches. One representative experiment from a total of three is shown.

We addressed these problems using a “reverse” approach. MM cell line INA-6 was chosen for selection of clones with stable and bright expression of EGFP, which were then electroporated with a stealth siRNA against EGFP in order to characterise the decline of green fluorescence (Fig. 3). Additionally, an expression plasmid for CD4Δ was also added to the electroporation mixture, and half of the transfected culture was subsequently purified by the CD4 microbead column procedure (i.e. yielding a relatively small number of strongly transfected cells) whereas the other half was only subjected to debris removal via OptiPrep gradient (i.e. yielding viable cells without any selection for “strong transfection”). FACS analysis of these cells showed that essentially the whole culture underwent a strong decline in EGFP intensity, which reached its nadir of less than 10% compared to mock-transfected control cells at about 4 days post-electroporation (Fig. 3). Notably, the extent of EGFP knockdown as well as its duration were virtually identical between both purification procedures, indicating that the siRNA had effectively and indiscriminately been introduced into the large majority of cells (Fig. 3). Conversely, electroporation of INA-6 cells with the EGFP-N3 plasmid usually results in no more than 30% efficiency (Table 1). The EGFP-range overlap between the siRNA-treated and the mock transfected cells at day three post-electroporation was 4.5%±2.8% (n = 3) and these events always formed a neat small peak right beneath the signal for the unaffected control cells (Fig. 3). The true nature of this “untransfected background” remains unclear, however. Although somewhat lower, this peak was also clearly visible in column-purified cell samples (Fig. 3), and we consider it very unlikely that any cells would have been transfected with the CD4Δ expression plasmid but not with the siRNA. These events might therefore rather represent dysfunctional cells or cell remnants which were not damaged badly enough to be excluded through forward/sideward scatter-based gating (and the preceding debris removal via OptiPrep), but without the structural integrity to perform siRNA-mediated degradation of EGFP. Taken together, these experiments clearly demonstrated that the electroporation of siRNAs into HMCLs can be nearly 100% effective at conditions when only a fraction of the cells are successfully transfected by expression plasmids (to be precise: by the EGFP and CD4Δ protein expression plasmids). This result potentially permits transient knockdown experiments in HMCLs on a considerably larger scale, so that more and/or different experiments (such as nuclear preparations) can be performed. For example, a typical electroporation using 3×10^7^ JJN-3 cells might yield a final tally of about 1×10^6^ cells via the column purification procedure, but should yield about 1.5×10^7^ cells if only debris removal is required. We therefore aimed to characterise this methodology in further detail.

### Short Hairpin RNA- versus Stealth siRNA-mediated ERK2 Knockdown in HMCLs

**Figure 4:**
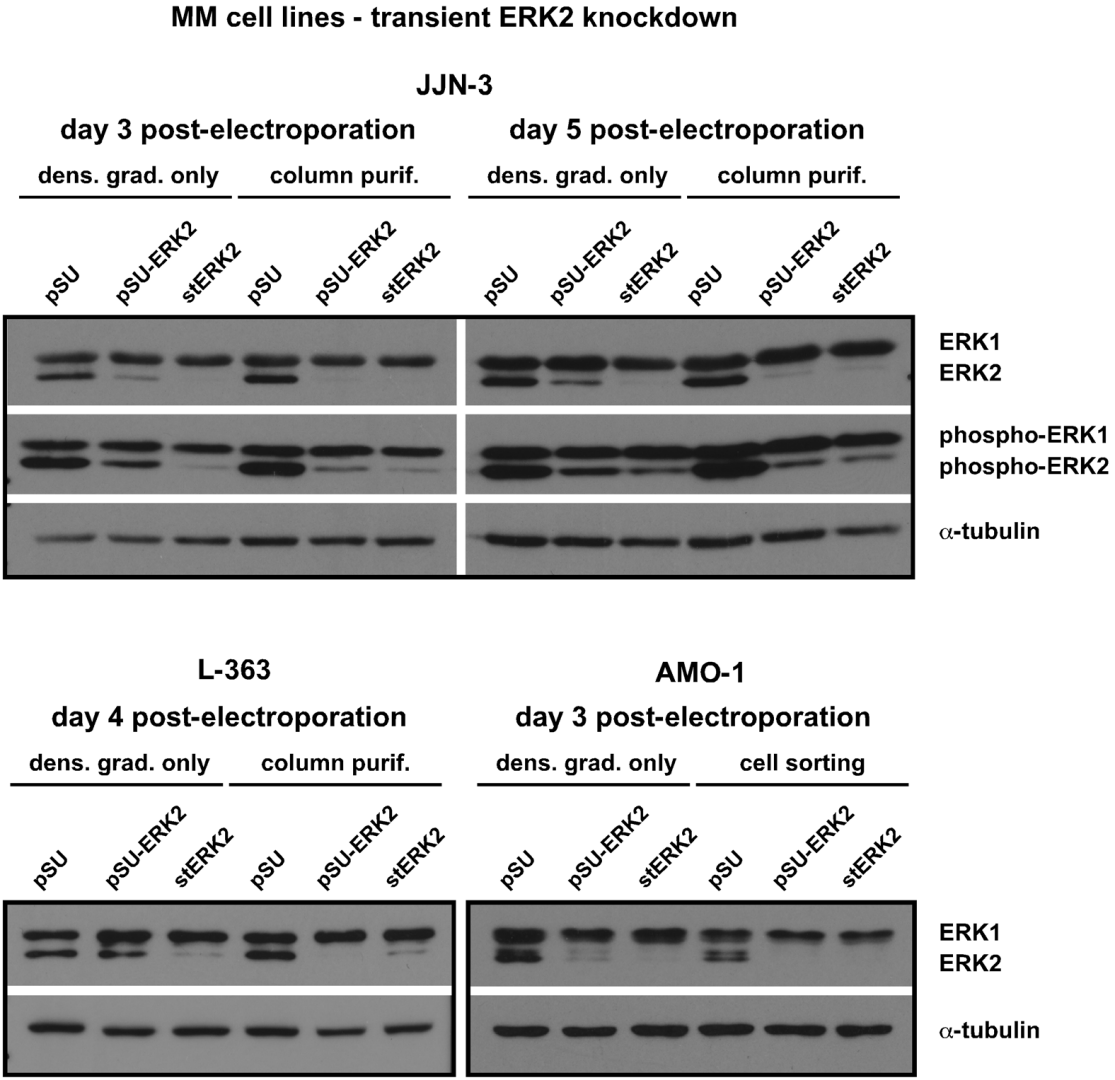
Knockdown efficiency in MM cells. Knockdown of ERK2 in different MM cell lines after transfection with either a short-hairpin expression vector (pSU-ERK2) or the “corresponding” target sequence synthesised as 25 bp stealth siRNA (stERK2; see Methods and [21]). At day 1 post-electroporation half of the transfected culture was subjected to the column purification method (also see Fig. 1b)–e)) (resp. cell sorting for AMO-1 cells) and the other half to debris removal only (also see Fig. 1f)). Cells were harvested for Western blotting at the times indicated. Empty pSUPER vector (pSU) transfected cells served as controls. The blots show that the ERK2 knockdown efficiency for stealth siRNA is virtually identical between cells that only underwent debris removal and those that were subjected to the column purification procedure. ERK2 knockdown using the short-hairpin expression vector was less efficient in debris-removal-only samples compared with their cognate column purification complements (see JJN-3, L-363). Representative experiments (JJN-3: n = 3; L-363: n = 2, AMO-1: n = 2) are shown. Anti-ERK1/2 antibody: CST.

We electroporated different HMCLs (AMO-1, JJN-3, L-363) using either a pSUPER-based shRNA expression plasmid against ERK2 (pSU-ERK2; [21]) or a 25 bp stealth siRNA (stERK2, based on the same core sequence as the shRNA, see Methods). pSUPER empty vector-transfected cells served as respective controls. At day 1 post-electroporation one half of each sample was subjected to the CD4 microbead column purification procedure (or to EGFP-based cell sorting for AMO-1 cells) and the other half simply to debris removal via OptiPrep density gradient. Cells were harvested for Western blotting and ERK1/2 staining up to day 5 post-electroporation (Fig. 4). Column purification or cell sorting yielded excellent knockdown results regardless of whether the shRNA expression plasmid or the stealth siRNA were used (Fig. 4). Conversely, differences between these reagents appeared if the cells were simply taken through the OptiPrep density gradient routine. Knockdown efficiencies for the stERK2 siRNA equalled those of the more complex purification procedures, but higher levels of ERK2 (or of its intrinsically activated form, phospho-ERK2) were observed for pSU-ERK2 transfections (Fig. 4). These experiments confirmed that electroporation using siRNA oligonucleotides represents a powerful and easy way to target the whole HMCL population. Somewhat surprisingly, the pSU-ERK2-mediated ERK2 knockdown in MM cells purified just via OptiPrep gradient was often still quite pronounced and certainly better than the presence of EGFP-negative cells (usually upwards of 50%) would have suggested. This effect was especially pronounced in AMO-1 cells, but was also noticeable in JJN-3 (Fig. 4), suggesting that in terms of electroporation efficiency the pSUPER plasmid (a 3.1 kb large derivative of the cloning vector pBluescript) might often rank closer to the much smaller siRNAs than to the EGFP protein expression vector (4.7 kb).

### Voltage Dependence of siRNA Electroporation in AMO-1 Cells

**Figure 5:**
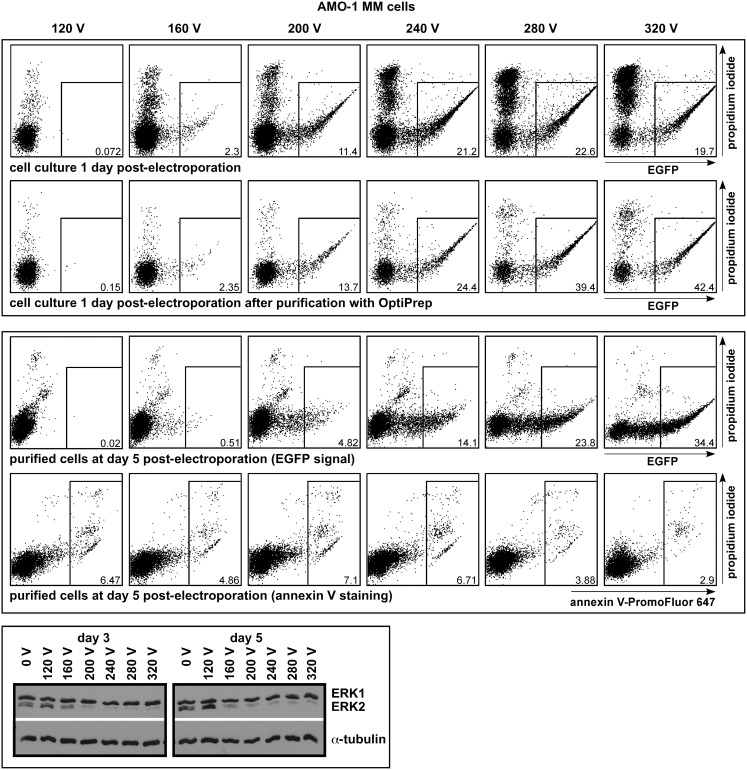
Voltage dependence of electroporation and knockdown efficiency in AMO-1 cells. Transfection of AMO-1 cells across a range of voltages using an expression vector for EGFP (pEGFP-N3) and a stealth siRNA against ERK2 (stERK2) in the electroporation mixture. Top panel: increases of the fractions of EGFP-expressing as well as of dead cells with higher voltages (top row). Cells taken in culture after OptiPrep-mediated debris removal reflect only the increase in transfection efficiency for the EGFP expression plasmid (bottom row). Middle panel: purified AMO-1 cells (those shown in the upper panel, bottom row) after culture for another 4 days. Top row: EGFP expression. Bottom row: annexin V-PromoFluor 647/PI staining. Even for the highest voltage used (320 V) the purified live cell fraction did not fare worse in subsequent culture than cells electroporated under milder conditions. Bottom panel: Western analysis of ERK2 knockdown at days 3 and 5 post-electroporation from the same cultures from which the FACS panels were derived. Efficient siRNA-mediated ERK2 knockdown was achieved at voltages significantly lower than required for the best levels of plasmid electroporation. However, a lower limit for successful knockdown was reached between the settings for 160 and 200 V. Shown is a representative experiment of two complete sets (Western blotting included). Anti-ERK1/2 antibody: CST.

In practice (i.e. for a defined and fixed set of electroporation parameters, such as electrode distance, volume, capacity, exponential decay-type pulse), voltage is perhaps the most important factor for transfection efficiency in electroporation [30]. Optimal conditions for any specific cell line are a trade-off between the number and signal intensity of affected cells on the one hand, and the ratio of living to dead cells on the other. Based on EGFP-expression from the pEGFP-N3 vector we found conditions of 270–310 V (at 960 µF with 4 mm cuvettes) most suitable to achieve acceptable rates of transfection and survival for those MM cell lines that are at all amenable to any such treatment (see Table 1). However, given that the use of siRNAs in such conditions resulted in virtually 100% transfection of the surviving cells, we tested if siRNA-mediated knockdown efficiency was also maintained at lower voltages, i.e. in conditions that exert lower experimental stress. AMO-1 cells were electroporated across a range of voltages with a mixture of the pEGFP-N3 vector (to determine transfection efficiency by EGFP expression) and the stERK2 siRNA (to determine transfection efficiency by ERK2 knockdown). One day post-electroporation all samples were cleaned of debris by OptiPrep treatment and then kept in culture for another 4 days, with samples for Western blotting taken at days 3 and 5 post-electroporation. At low voltages (here: 120 V) virtually no cell death ensued, but neither were any EGFP-positive cells visible. At the high end (here: 320 V) the transfection efficiency was about 20%, but a large number of cells did not survive the procedure (Fig. 5, top panel – upper row). After debris removal the increase in EGFP expression within the live cell fraction with rising voltages was clearly visible (Fig. 5, top panel – lower row), although this came at the expense of lower absolute numbers of live cells (not shown). Staining for ERK2 showed that about the same level of depletion was achieved for voltages of 200 and higher (Fig. 5, lower panel). 200 V corresponded to the setting at which EGFP-positive cells began to appear in substantial quantities (11.4% in the exemplary experiment shown in Fig. 5 (upper panel)). No differences in ERK2 knockdown efficiency were observed between cells harvested at days 3 and 5 post-electroporation, although it remains possible that the effect might fade faster for lower voltage settings if still longer-term cultures were evaluated. These experiments show that in siRNA electroporations of MM cells milder voltage settings are permissible, and that an “imperfect” indicator – such as the EGFP-N3 plasmid used here – can be used to demarcate a suitable lower limit. Such considerations are important because – as with any other transfection technique – confounding aspects of the effects of electroporation-based methods on the cellular assays performed must always be accounted for [31]. Of note, annexin V/PI-staining of the cultured AMO-1 cells at day 5 post-electroporation (i.e. day 4 post-OptiPrep purification) showed no substantial differences in apoptosis between the different voltage settings, showing that viable cells isolated at day 1 post-electroporation recovered well even for the highest voltages applied (Fig. 5, middle panel – lower row).

### General Applicability of Electroporation Procedures for MM Cells

**Figure 6:**
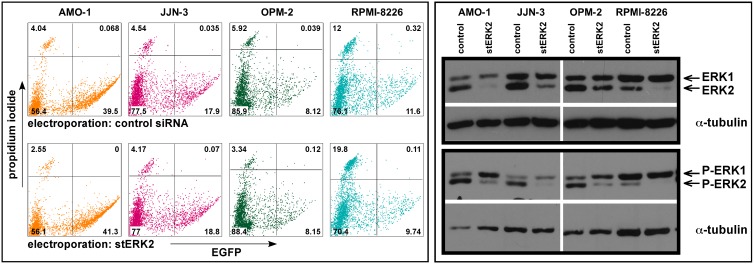
Electroporation and knockdown efficiencies in “easy-to-transfect” vs. “hard-to-transfect” MM cell lines. Left-hand panel: MM cell lines were electroporated with an expression vector for EGFP (pEGFP-N3; 10 µg/ml) and stealth siRNAs against either ERK2 (stERK2; 3 µM) or against no specific target (control; 3 µM). The FACS-measurements represent the cell cultures at day 1 post-electroporation after debris removal with OptiPrep. Right-hand panel: Knockdown of ERK2 and intrinsic levels of phospho-ERK2 (cells from the cultures represented on the left were harvested at day 3 post-electroporation for Western blotting). Good knockdown of ERK2 and lowered levels of phospho-ERK2 were found for all four MM cell lines tested. Shown is a representative experiment of two complete sets (Western blotting included). Anti-ERK1/2 antibody: Santa Cruz Biotechnology.

Based on EGFP expression we distinguish the MM cell lines available in our laboratory as either easy to electroporate (i.e. generally yielding transfection rates >10%, e.g. AMO-1, INA-6, JJN-3, L-363, MM.1S, MOLP-8, U-266), or as hard to electroporate (NCI-H929, OPM-2, RPMI-8226; Table 1). We therefore tested if siRNA-mediated ERK2 knockdown was also productive in the latter cell lines, which we consider unsuitable for our plasmid-based knockdown protocols. MM cells were electroporated with either the ERK2 stealth siRNA or an unspecific control stealth siRNA as well as with the pEGFP-N3 marker plasmid, and dead cells were removed via OptiPrep gradient at day one post-electroporation (Fig. 6, left panel). ERK2 knockdown and reduction of intrinsic phospho-ERK2 levels were subsequently determined by Western blotting. The extent of ERK2 knockdown in OPM-2 and RPMI-8226 cells was roughly on a par with levels achieved in AMO-1 and JJN-3 cells (Fig. 6, right panel), showing that MM cell lines that only display low EGFP expression plasmid penetrance can still successfully be used in siRNA electroporation experiments. Similar to the dependence on voltage discussed above, a modicum of EGFP expression suffices to indicate conditions suitable for successful electroporation with siRNAs.

### General Considerations on the Use of Electroporation-based Knockdown Protocols with MM Cell Lines

RNA interference experiments are powerful means to conduct loss-of-function analyses, be they in order to complement experiments with pharmacological inhibitors or to analyse targets for which suitable inhibitors have not yet been developed. Because MM cell lines generally grow in suspension or at best semi-adherently, they belong among the harder-to-manipulate cell types by transient transfection methodologies. Even though a number of papers either deal with this issue or report MM cell transfection in their methods chapter [14], [15], [16], [17], [18], [19], [20], a robustly workable and widely-used protocol has not yet emerged. Our analyses of MM cell electroporability with siRNAs were therefore not only intended to address the purely functional aspects, but also to judge whether such a protocol could i) easily be performed and replicated by other researchers in their laboratories, ii) prove cost-effective, and iii) could be applied to generate sufficient numbers of transfected cells to conduct experiments that require substantial amounts of material, such as, for example, nuclear preparations (i.e. cell numbers in the range of several millions rather than hundreds of thousands). Regarding ease-of-performance electroporation certainly fits the bill, since all that is required is an electroporation device in addition to standard cell culture laboratory equipment. A very basic instrument, capable of providing an exponential decay-type current is sufficient. It has been described for other cell lines that electroporation protocols employing more than one electric pulse [32] or continuous square pulsing at two different field strengths [30] can result in superior rates of transfection, as determined by EGFP expression. Although we have occasionally tested square pulse-type current deliveries we have not found this to consistently achieve (much) better rates in EGFP-expressing MM cells than a single standard exponential decay-type pulse. Regarding electroporation of siRNAs such differences become moot anyway, as we have shown here that the whole surviving MM cell fraction is successfully being transfected. In terms of cost effectiveness, the main expense is the need to buy siRNAs. Because different sequences and different targets may yield different knockdown results (depending, amongst other factors, on protein expression levels and the rate of protein turnover) we would recommend to titrate the effects of a new siRNA employed in electroporation. Based on hands-on experience with about 15 different siRNAs and targets a concentration of between 1–3 µM in the electroporation mix is usually required. 80 nM of a custom-built siRNA will currently (2014) cost about 450 € and provides enough reagent for 50 electroporations at 3 µM concentration and 500 µl of cell suspension (the standard volume we use in 4 mm electroporation cuvettes). Using 2 mm cuvettes it is sufficient for 125 electroporations à 3 µM/200 µl. These numbers are certainly no worse than, for example, expenses incurred for many antibodies used in a world-wide standard procedure such as Western blotting. Finally, an especially appealing aspect of the protocol is the ease with which it can be scaled up. Densities of up to 5×10^7^ MM cells/ml are entirely permissible, which for an electroporation in 200 µl volume translates into 1×10^7^ cells of which about 3–5×10^6^ should be recoverable alive after OptiPrep cleaning at day 1 post-electroporation. This is sufficient for multiple parallel measurements, such as cell death determinations, proliferation assays, Western analyses etc. at various time-points, and obviously more electroporations can effortlessly be performed if still higher cell numbers are needed. Taken together, we believe that the siRNA electroporation procedure detailed here can confidently be employed in any laboratory devoted to MM cell research.

## Supporting Information

Figure S1 **Electroporation of RPMI-8226 cells with a 6-FAM-labelled siRNA oligonucleotide.** Top: Fluorescence of RPMI-8226 cells electroporated with the siERK2-6-FAM oligonucleotide (green curve) in relation to mock transfected cells (blue curve) at different time points post-electroporation (p.e.). Bottom: Western analysis for ERK2 knockdown at days 3 and 5 post-electroporation.
(TIF)
