# Supplementary figures and images for: Efficient Transient Transfection of Human Multiple Myeloma Cells by Electroporation – An Appraisal
Source: PLoS One. 2014 Jun 5;9(6):e97443. doi: 10.1371/journal.pone.0097443 (PMC4047019; doi:10.1371/journal.pone.0097443)

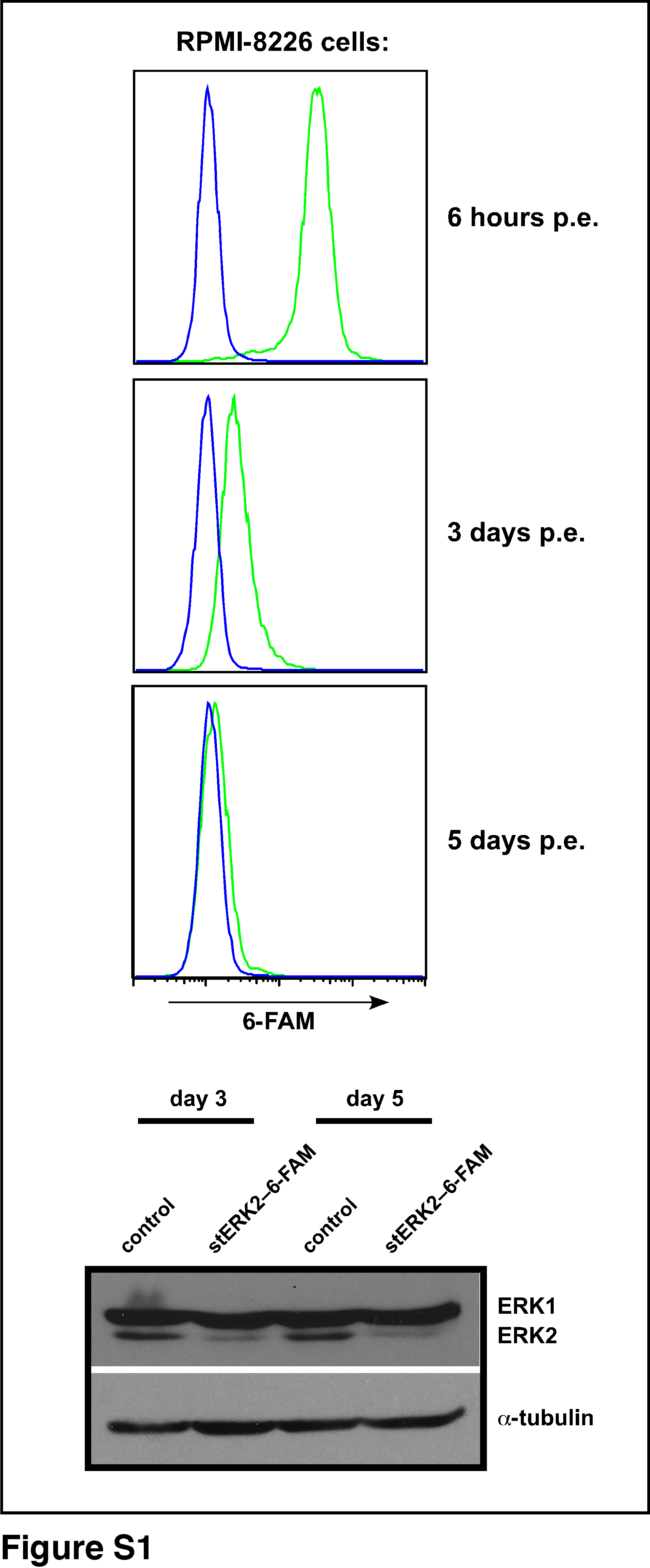

Supplement: Figure S1 — Electroporation of RPMI-8226 cells with a 6-FAM-labelled siRNA oligonucleotide. Top: Fluorescence of RPMI-8226 cells electroporated with the siERK2-6-FAM oligonucleotide (green curve) in relation to mock transfected cells (blue curve) at different time points post-electroporation (p.e.). Bottom: Western analysis for ERK2 knockdown at days 3 and 5 post-electroporation. (TIF) [file pone.0097443.s001.tif]
